# Impaired heart rate variability predicts clinical deterioration and progressive organ failure in emergency department sepsis patients

**DOI:** 10.1186/cc10644

**Published:** 2012-03-20

**Authors:** R Arnold, G Green, A Bravi, S Hollenberg, A Seely

**Affiliations:** 1 Cooper University Hospital, Camden, NJ, USA

## Introduction

Emergency department (ED) sepsis patients without overt shock have a high incidence of clinical deterioration after admission. Heart rate variability (HRV) is decreased in severe sepsis. The objective was to determine the ability of a panel of HRV indices to identify physiologically stable ED sepsis patients who will develop worsening organ failure. We hypothesized that patients meeting the outcome of progressive organ failure will have decreased HRV on initial presentation.

## Methods

We performed a prospective observational study of adult ED patients admitted to the hospital for infection and treated with i.v. antibiotics. Patients in overt shock (vasopressor requirement or mechanical ventilation) at enrollment or with the inability to provide written informed consent were excluded. A panel of HRV indices was assessed over a 2-hour ED period using CIMVA (continuous individualized multiorgan variability analysis) software including standard deviation (SD), LF/HF ratio, Poincare SD, sample entropy, wavelet AUC, detrended fluctuation analysis (DFA), correlation dimension, and the Lyapunov exponent. Patients were followed to assess the occurrence of the primary outcome of increased organ failure (SOFA score increase greater than 1 point at 24 hours), mechanical ventilation, vasopressor use, or in-hospital mortality.

## Results

We enrolled 105 ED sepsis patients. Twenty patients were removed due to nonsinus cardiac rhythm or poor data quality of the telemetry signal. Complete HRV assessment was performed on 81 subjects with 17 patients removed who developed shock in the ED. The primary outcome was met in 44% (28/64) of the cohort. On HRV assessment, outcome patients had a lower LF/HF ratio (1.47 vs. 3.11, *P *= 0.009) and DFA (0.65 vs. 0.94, *P *= 0.04) compared with stable patients with no differences in other HRV indices. The overall mortality rate was 15%. Compared to stable patients, outcome patients had no difference in age, initial heart rate, systolic blood pressure, or serum lactate with similar initial SOFA scores that were higher at 24 hours (1.0 vs. 3.0), a higher ICU transfer rate (62 vs. 20%, *P *< 0.001) and increased ICU length of stay.

## Conclusion

While standard physiologic parameters in the ED were unable to differentiate sepsis patients who developed increased organ failure, a decreased LF/HF ratio and DFA, measurements of variability representing physiologic reserve, was associated with impending deterioration. The ability of decreased HRV to predict clinical outcomes in a high-risk yet physiologically identical population at presentation supports the need for continued studies into the predictive role of HRV assessment in the ED to supplement clinical decision-making in sepsis patients.

